# Biliary Calculi

**Published:** 1875-10

**Authors:** 


Biliary Calculi Extracted Through the Abdominal Walls.—M. Brousson, of Nimes, describes the following case: A patient was suffering from a painful, ill-defined tumor, situated in the right flank, between the spine of the ileum and umbilicus. The tumor was taken to be a suppurating ovary, and was opened with the caustic Vienna-paste. The tumor was found to be due to an accumulation of biliary calculi, of which no less than forty were removed. In three months the patient had recovered. *Medical Record.*

